# Effect of the severity of liver dysfunction on the minimum alveolar concentration of sevoflurane responding to an electronic stimulation in cirrhotic patients

**DOI:** 10.1186/s12871-016-0260-8

**Published:** 2016-10-18

**Authors:** Yan Yin, Hong Xiao, Jirimutuya Han, Weiyi Zhang, Jianguo Cheng, Tao Zhu

**Affiliations:** 1 Department of Anaesthesiology, West China Hospital, Sichuan University, Chengdu, Sichuan Province 610041 China; 2 Department of Anaesthesiology, Chengdu Women’s and Children’s Central Hospital, Chengdu, Sichuan Province 610031 China; 3 Departments of Pain Management and Neurosciences, Lerner Research Institute and Anaesthesiology Institute, Cleveland Clinic, Cleveland, OH 44195 USA

**Keywords:** Sevoflurane, Minimum alveolar concentration, Bispectral index, Liver cirrhosis, Minimal hepatic encephalopathy

## Abstract

**Background:**

It has been observed that patients with liver dysfunction need lower dose anesthetic compared patients with normal liver function. The minimum amount of volatile anesthetic to achieve an optimal depth of anesthesia for these patients is still unclear. In this study, Minimum alveolar concentration (MAC) of the sevoflurane was determined using an electric stimulation and the effect of severity of liver dysfunction on the MAC was observed in cirrhotic patients.

**Methods:**

Thirty patients undergoing upper abdominal surgery were divided into the following groups: group N (normal liver function), group A (Child-Pugh grade A) and group B (Child-Pugh grade B-C). Neuropsychological tests were performed before surgery. We measured MAC_electric_ (minimum alveolar concentration that prevents movement in response to an electric stimulation in 50 % of patients) of sevoflurane in cirrhotic patients with liver dysfunction using an electrical stimulation of 80 mA at 50 Hz, and analyzed factors that associated change of MAC.

**Results:**

According to the neuropsychological tests, there were 7 and 4 patients with minimal hepatic encephalopathy in Groups B and A, respectively. MAC_electric_ in cirrhotic patients with liver dysfunction decreased significantly compared to that in healthy liver patients (1.51 ± 0.16 vol. %, 1.33 ± 0.14 vol. % and 1.17 ± 0.13 vol. % in Group N, A and B, respectively), while MAC_electric_ was comparable between the cirrhotic patients with different Child-Pugh grade. The Alanine Aminotransferase (ALT) and baseline values of bispectral index (BIS) were risk factors associated with the lowering of MAC_electric_ (*p* < 0.05).

**Conclusion:**

MAC_electric_ of sevoflurane in cirrhotic patients was significantly lower than that of patients with a healthy liver. The severity of liver dysfunction had no effect on the MAC_electric_ of sevoflurane in cirrhotic patients.

**Trial registration:**

This study has been registered in the Chinese Clinical Trial Register in August 3, 2011 (No. ChiCTR-TRC-11001507).

## Background

The minimum alveolar concentration (MAC) of anesthetic that prevents movement in 50 % of subjects in response to a noxious stimulus [1], is used to measure the capability of volatile anesthetics to immobilize patients who are exposed to noxious stimulation. However, it has been shown that MAC may be affected by many pathophysiological conditions and disease states in animals and patients [2–7]. The liver is a vital organ involved in drug distribution, metabolism, and elimination. Although the lifespan of people with asymptomatic liver cirrhosis is not different from that of healthy people [8], the perioperative morbidity and mortality increase significantly in patients with liver cirrhosis [9, 10].

It has been shown that there is an association between excessive depth of anesthesia and poor postoperative outcomes, especially in high-risk patients [11, 12]. Providing safe and effective anesthesia in patients with cirrhosis and liver dysfunction has been a daunting challenge for most of the anesthesiologists. Various clinical and animal studies suggest that volatile anesthetic requirement is decreased in subjects with liver dysfunction and is correlated with the severity of liver dysfunction [13–15]. However, to our knowledge, the MAC to a noxious stimulus of a volatile anesthetic has not been previously evaluated in cirrhotic patients. Thus, we aimed to determine the MAC values (MAC_electric_) of sevoflurane in cirrhotic patients by using a supramaximal electric stimulation and evaluate the effect of severity of liver dysfunction on MAC_electric_. We provided data that shed light on the impact of liver dysfunction on the requirement of volatile anesthetics in humans.

## Methods

The research protocol was approved by the Institutional Review Board (IRB) of West China Hospital. Patients undergoing selective upper abdominal surgery from September 2012 to June 2013 were screened for this study. Excluded from enrollment were patients with: 1) difficult airway; 2) illiteracy who could not follow instructions; 3) a body mass index (BMI) ≥30 kg/m^2^;4) taking chronic sedatives and alcoholics; and 5) serious diseases other than liver diseases, such as cardiovascular, respiratory, endocrine system conditions. For those who met the inclusion criteria and were willing to participate, written informed consent was obtained from each patient. A total 30 male patients were enrolled and divided into three groups according to their liver function status: Group N, patients with normal liver function undergoing upper abdominal surgery (*n* = 10); Group A, patients with cirrhotic liver of Child-Pugh grade [16] A (score 5 or 6) undergoing hepatectomy (*n* = 10); Group B, patients cirrhotic liver of Child-Pugh grade B or C (score ≥ 7) undergoing Minch devascularization and splenectomy or liver transplantation (*n* = 10). Diagnosis of cirrhosis was based on clinical, biochemical, ultrasonographyor computed tomography scan and liver histological findings if available.

All patients were screened for minimal hepatic encephalopathy (MHE) according to the Number Connection Test A (NCT-A) and Digit Symbol Test (DST) 1 day before the surgery. A test was considered abnormal when the score was beyond ±2 SD from the score in the age and education-matched control group [17, 18]. MHE was diagnosed if one or two tests were abnormal.

All patients received an identical anesthetic technique and monitoring devices, consisting of continuous electrocardiography, pulse oximetry, the arterial line for blood pressure monitoring, nasopharyngeal temperature and bispectral index (BIS) (BeneView T8, Cayman Mindray Medical Electronics Co., Ltd, Shenzhen, China). Expired sevoflurane and CO_2_ concentrations (ETCO_2_) were continuously monitored using infrared analyzers (M1026B, Philips Medizin System Boeblingen GmbH, Boblingen, Germany). None of the patients received premedication. Anesthesia was induced using a mask with 8 % sevoflurane in 6 L/min oxygen, and a Proseal laryngeal mask was inserted when patients’ eyelash reflex was lost, and the end-tidal sevoflurane was greater than 3 %. The oxygen flow rate was then adjusted to 3 L/min, and normocapnia (ETCO_2_ 35–45 mmHg) was maintained. Manual ventilation was administrated if necessary.

The MAC_electric_ values were estimated using the method previously described [19, 20]. An electrical stimulation of 80 mA at 50 Hz (Neurostim T4, HSE, Germany) on the ulnar aspect of the forearm at the midpoint was performed for 15 s or until a purposeful movement was observed [21]. Purposeful movement was defined as a substantial movement of the head or extremities following the electric stimulation. Local muscle contractions at the stimulation site and related finger flexion constitute slight movement and are not regarded as purposeful movement. The end-tidal sevoflurane concentration of the first patient in Group N began from1.71 vol. % (MAC that produces immobility exposed to a skin incision) [4]. When patients exhibited purposeful movement, the end-tidal concentration of sevoflurane was increased by 0.2 %, and the subjects were retested after 10 min of re-equilibration. If the patient did not move, the end-tidal concentration of sevoflurane was decreased by 0.2 %. The MAC_electric_ of each patient was calculated as the value halfway between the end-tidal concentrations that prevented or allowed purposeful movement in response to the electrical stimulation. The next patient’s initially end-tidal sevoflurane concentration was set at the MAC_electric_ obtained from the preceding patient. The same method was used to determine the MAC_electric_ for each patient’s. The MAC_electric_ for each group was calculated as the mean of the ten patients.

Supramaximal stimulation is required to determine MAC. Since previous studies have shown that pain threshold increases in patients with liver dysfunction, we used parameters that were determined by our pilot data that showed the electric pain tolerance threshold were 41.9 ± 15.4, 50.5 ± 10.39 and 58.9 ± 2.08 mA in healthy, Child grade A, and Child grade B patients (*n* = 10) respectively. Initially, we used 60 mA as the highest current to measure pain thresholds in the awaken patients and pain tolerance over 60 mA was recorded as 60 mA. In such cases, 60 mA was no longer supramaximal stimulation. Since 80 mA at 50 Hz has been confirmed to cause no damage to tissues in animals and healthy volunteers, we chose to use 80 mA, instead of 60 mA, to determine MAC in this study.

All data were analyzed using the IBM SPSS Version 21.0. All original data were first analyzed with Shapiro-Wilk test to examine whether data were in normal distraction. Data were presented as mean ± standard deviation (SD). The data without normal distribution were presented as medians with maximum and minimum.

The results of the three groups were compared using one-way ANOVA. Bonferroni or Dunnett T3 tests were used to analyze the statistical difference between two groups, according to the results of homogeneity test of variance. Student’s *t*-test was used to compare the data between patents with or without MHE. The Wilcoxon’s signed rank test was used for data in non-normal distribution. Related factors were analyzed using Spearman correlation analysis and multiple linear regressions with a stepwise selection method. *p* < 0.05 was considered statistically significant.

## Results

All patients completed the study. The demographic data of patients are reported in Table 1. The results of laboratory examination and the blood gas analysis are shown in Tables 2 and 3 respectively. Compared to patients with normal liver functions, the values of hepatic function and blood coagulation parameters were significantly abnormal in patients with cirrhotic livers.

**Table 1:** Demographic data

	Group N	Group A	Group B	*P-*value
Age (Years)	47.1 ± 10.73	47.9 ± 8.29	40.1 ± 8.39	0.133
BMI (kg/m^2^)	24.04 ± 1.40	22.6 ± 3.62	21.44 ± 3.03	0.142
Surgery type				
Stomach	5			
Gallbladder	5			
Hepatectomy		10		
Minch devascularization and splenectomy			5	
Liver transplantation			5	

Data were presented as the means and SDs

*Abbreviations: BMI* body mass index

**Table 2:** Biochemical markers before surgery

	Group N	Group A	Group B	*P*-value
TBIL (μmol/l)	9.8 (5.7,24.2)	16.85 (12.6,26.6)	42.9 (18.7,620.3)^a,b^	<0.001
DBIL (μmol/l)	3.05 (2.1,7.9)	7.25 (5.2,9.9)	15.85 (7.9,453.9)^a, b^	<0.001
IBIL (μmol/l)	6.65 (3.5,16.3)	10.50 (6.8,16.7)	20.1 (6.4,166.4)^a^	0.003
ALT (IU/l)	21.5 (16,56)	50.0 (22,156)	25.5 (10,80)^a^	0.016
AST (IU/l)	24.5 (19,37)	41.5 (23,142)	37.5 (10,119)^a^	0.014
ALB (g/l)	41.83 ± 3.83	39.13 ± 2.8	29.63 ± 4.12^a, b^	<0.001
LDH (IU/l)	144.5 (105,247)	162.5 (56,312)	148 (105,197)	0.451
BUN (μmol/l)	6.07 (4.04,67)	5. 1 (3.59,6.72)	4.0 (3.22,11.38)	0.192
SCr (μmol/l)	91.8 (57.8,101.9)	79 (66.2,96.1)	75.05 (57,138)	0.81
Ammonia (μmol/l)	35.5 ± 20.42	49.6 ± 26.81	57.0 ± 25.99	0.158
PT (s)	10.7 (10.4,12)	12.65 (11.3,14.5)	17.4 (13.7,23.4)^a, b^	<0.001
APTT (s)	27.45 (22.1,32.5)	30.05 (25.9,36.1)	41.45 (2.07,59)^a^	0.001
FIB (g/l)	2.83 (1.71,4.25)	2.78 (1.78,6.16)	1.22 (0.63,3.64)^a^	0.015

Data with normal distribution were presented as the means and SDs. Data without normal distribution were presented as medians with maximum and minimum. The *P*-values were derived from ANOVA or Wilcoxon’s signed rank test. Bonferroni or Dunnett T3 tests were used for *post hoc* paired comparisons

*Abbreviations: TBIL* total bilirubin, *DBIL* direct bilirbubin, *IBIL* indirect blilirubin, *ALT* alanine aminotransferase, *AST* aspartame aminotransferase, *ALB* albumin, *LDH* lactate dehydrogenase, *BUN* urea nitrogen, *SCr* serum creatinine, *PT* prothrombin time, *APTT* activated partial thromboplastin time, *FIB* fibrinogen

^a^Group B vs. Group N, *P* < 0.05

^b^Group B vs. Group A, *P* < 0.05

**Table 3:** Blood gas analysis before surgery

	Group N	Group A	Group B	*P-*value
pH	7.44 (7.36,7.49)	7.42 (7.36,7.5)	7.39 (6.97,7.53)	0.459
PaO_2_ (mmHg)	193.5 (156,317)	198.7 (158,251)	194.2 (112,271)	0.898
PaCO_2_ (mmHg)	33.81 ± 3.07	34.97 ± 8.17	37.01 ± 3.63	0.426
BE (mmol/l)	−1.07 ± 2.47	−1.66 ± 2.81	−1.83 ± 9.14	0.952
HCO_3_^−^ (mmol/l)	22.49 ± 1.67	22.94 ± 2.9	22.45 ± 6.55	0.96
K^+^ (μmol/l)	3.37 ± 0.5	3.43 ± 0.32	3.63 ± 0.55	0.412
Na^+^ (μmol/l)	141.6 ± 2.72	141.26 ± 2.86	137.9 ± 3.6^a^	0.022
Ca^2+^ (μmol/l)	1.07 ± 0.07	1.08 ± 0.38	1.11 ± 0.06	0.233
Hb (g/l)	110.1 ± 39.71	100.7 ± 41.73	95.9 ± 34.72	0.408
Hct (%)	37.1 ± 6.52	33.3 ± 8.35	29 ± 10.71	0.134

^a^Group B vs. Group N, *P* < 0.05

Results of neuropsychological tests and BIS values before the study are showed in Table 4. These values were used to screen for MHE. Patients with Child-Pugh score over 6 required longer time to finish NCT-A and achieved lower DST scores compared to patients with healthy liver (*p* < 0.05). Based on the results of NCT-A and DST, there were 4 and 7 patients with MHE in Groups A and B, respectively. The BIS values of Groups A and B were significantly lower than those of Groups N. The BIS values in cirrhotic patients with MHE were significantly lower than those in patients without MHE (92.71 ± 3.82 vs. 95.88 ± 1.19, *p* = 0.025), when patients were awake.

**Table 4:** Neuropsychological test results and BIS value before surgery

	Group N	Group A	Group B	*P*-value
NCT-A (s)	39.86 ± 10.0	62.29 ± 26.9	73.64 ± 32.1^a^	0.017
DST	11.55 (9.8,17.1)	10.25 (6.1,15.1)	8.5 (5.1,12.2)^a^	0.015
BIS	97 (97,99)	97 (94,97)	94 (87,97)^a^	<0.001
MHE	0	4	7	

NCT-A: The Number Connection Test A (Range of normal values: 10–66 s). DST: Digit Symbol Test, the scores were obtained in 90 s according to Wechsler Intelligence Scale for Adult-Chinese Revised (WAIS-RC). BIS: the bispectral index. MHE: minimal hepatic encephalopathy, which was diagnosed if one or both the two neuropsychological tests were abnormal

^a^Group B vs. Group N, *P* < 0.05

The vital sign and BIS values measured continuously during the study, which showed in Table 5. MAP, HR, and SPO_2_ were kept stable throughout the study in three groups, although MAP and HR significantly increased in patients of Group A compared to patients of Group N and B when performed the electric stimulation.

**Table 5:** BIS value and vital signs during the study

	Group N	Group A	Group B	*P*-value
Before anesthesia				
HR (beat/min)	76 (63,92)	83.5 (71,122)	82.5 (65,118)	0.15
SpO_2_ (%)	98.3 ± 1.06	97.7 ± 1.25	97.3 ± 1.83	0.3
MAP (mmHg)	82.7 ± 14.22	87.2 ± 32.05	78.9 ± 9.04	0.104
BIS	97 (97,99)	97 (94,97)	94 (87,97)^a^	<0.001
Time of MAC_electric_1				
HR (beat/min)	63.6 ± 7.20	76.2 ± 7.24^a^	71.4 ± 11.21	0.012
SpO_2_ (%)	100 (99,100)	100 (99,100)	100 (99,100)	0.631
MAP (mmHg)	65 (54,85)	75 (61,89)	64 (60,79)^a^	0.028
BIS	46.4 ± 6.69	49.1 ± 9.36	46.4 ± 6.59	0.665
Time of MAC_electric_2				
HR (beat/min)	65.9 ± 7.05	80.7 ± 7.10^a^	73.7 ± 11.26	0.003
SpO_2_ (%)	100 (99,100)	100 (99,100)	100 (99,100)	0.631
MAP (mmHg)	67 (57,90)	85 (77,103)^a^	65.5 (62,84)^b^	0.001
BIS	52.3 ± 7.32	56.1 ± 9.13	51.2 ± 11.59	0.519

Time of MAC_electric_ 1: The time when the lowest end-tidal concentration of sevoflurane that prevented purposeful movement was determined

Time of MAC_electric_2: The time when the highest end-tidal concentration of sevoflurane that did not prevent purposeful movement was determined

*Abbreviations: HR* heart rate, *SpO2* pulse oximetry, *MAP* mean arterial pressure, *BIS* bispectral index

^a^Compared with Group N, *P* < 0.05

^b^Compared with Group A, *P* < 0.05

**Fig. 1:**
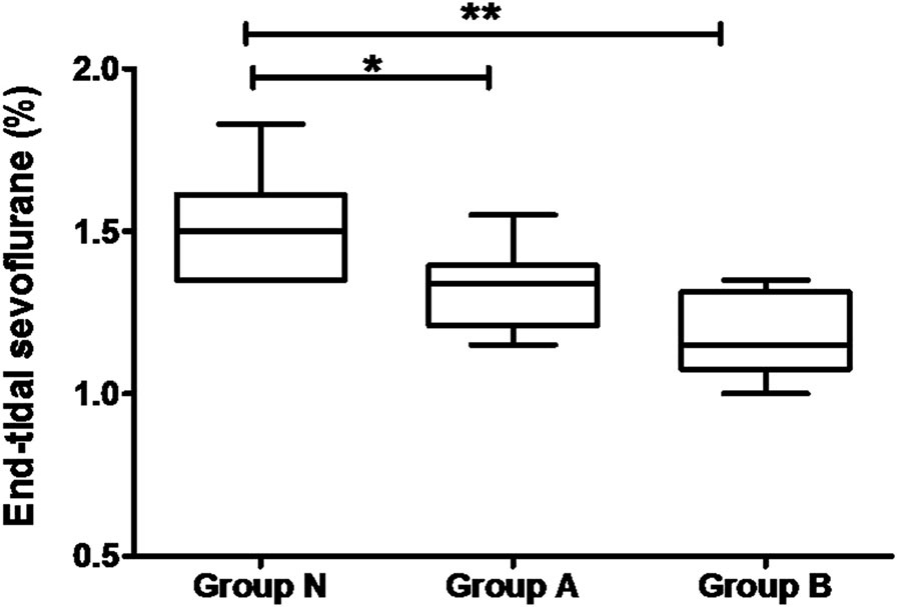
MAC_electric_ of three groups. Reduced MAC_electric_ in patients with liver dysfunction. The MAC_electric_ of each patient was calculated as the value halfway between the end-tidal concentrations of sevoflurane that prevented or allowed purposeful movement in response to the electrical stimulation (80 mA 50 Hz for 15 s). Group MAC_electric_ values are expressed as median ± maximum and minimum. The MAC_electric_ values in Groups B and A were significantly lower than that of Group N patients with normal liver function parameters (** *p* < 0.001 and * *p* = 0.042)

The results of MAC_electric_ are presented in a box- and -whisker plot (Fig. 1). The means of MAC_electric_ in patients with liver dysfunction were significantly lower than that of patients with normal liver functions (1.51 ± 0.16 vol. % for Group N, vs. 1.33 ± 0.14 vol. % or1.17 ± 0.13 vol. % for Group A and B, respectively, *p* < 0.05). The difference between Groups A and B were not statistically significant (*p* > 0.05). The mean MAC_electric_ was lower in patients with MHE compared with those without it (1.19 ± 0.12 vol. % vs. 1.32 ± 0.14 vol. %, *p* = 0.028).

To examine the factors that may affect MAC_electric,_ Spearman analysis were used to analyze the correlation between a number of factors and the change of MAC_electric._ These factors were Child-Pugh score; NCT-A, DST, BIS index, total bilirubin (TBIL), direct bilirubin (DBIL), alanine aminotransferase (ALT), aspartame aminotransferase (AST), and albumin. Upon multivariate analysis, ALT and BIS values before surgery were significantly correlated with MAC_electric_ among the groups. The regression equation (MAC_electric_ = −0.003 ALT +0.053 BIS) was statistically significant (F = 3.845, *p* = 0.006).

## Discussion

The end-tidal concentration of a volatile agent is an essential component of the concept of MAC, which is widely used as an index of anesthetic potency of a volatile anesthetic. In this work, we found that the mean MAC_electric_ was significantly reduced in patients with liver dysfunction (Child- Pugh grade A, and grade B or C) compared to patients with normal liver function (Group B vs. Group N, *p* < 0.001; Group A vs. Group N, *p* < 0.05). This observation is consistent with published data [13–15]. We have previously reported that the sevoflurane MAC values in animals with chemically-induced liver fibrosis were significantly lower than that in animals with normal liver function [14]. However, we did not found statistically significant difference between patient groups with different levels of liver dysfunction (Group A vs. Group B, *p* > 0.05). This is likely due to the fact that the study was under powered and limited by the relatively small sample size. Both Wang [13] and Kang et al. [15] report that the severity of liver dysfunction influenced the requirements of volatile anesthetic in orthotopic liver transplantation patients to maintain preset anesthetic depths, as monitored by a target BIS. In addition to sample size, other factors may also contribute to this discrepancy, such as patient characteristics and use of different score systems for the severity of liver diseases. Nonetheless, it is clear that liver dysfunction status plays a significant role in the requirement of volatile anesthetics.

The mechanisms of reduced volatile anesthetic requirement in cirrhotic patients are poorly understood. We demonstrate that ALT and BIS were the risk factors associated the decrease of MAC_electric_. These results support the notion that these parameters may be used as important predictors of decreased requirement of sevoflurane in these patients. Accumulating evidence supports that inhaled anesthetics exert their effects by acting on ion channels and receptors in neurons and altering synaptic transmission in the central nervous system [22–24]. For example, the MAC of halothane in children with congenital cerebral palsy or severe mental retardation was lower than that in normal children [6]. The most widely recognized relationship between the liver and the brain is exemplified by hepatic encephalopathy (HE), which is broadly divided into overt HE and MEH, which is a subclinical HE without clinically overt symptoms. However, MEH has been associated with increased mortality, the risk of hospitalization, and increased caregiver burden [25]. MHE can be diagnosed using hypersensitive neuropsychological tests including NCT-A and DST [26]. In this study, there were 40 and 70 % patients with MHE in Group A and Group B respectively, which is consistent with the previously reported prevalence of MHE in cirrhosis [27, 28]. The MAC_electric_ was lower in cirrhotic patients with MHE compared those without it.

Previously studies have demonstrated that BIS is a useful tool not only in monitoring the depth of anesthesia [12, 13] but also in diagnosing MHE, HE and grading HE [29, 30]. Consistent with these observations, our results further showed that BIS values in cirrhotic patients with a Child-Pugh score over 6 were significantly lower than those patients with a healthy liver (Table 4) even before the initiation of anesthesia. It may be considered as a predictor of reduced MAC in response to electric stimulation in cirrhotic patients. Furthermore, cirrhotic patients with MHE have lower BIS values than those cirrhotic patients without MHE. These findings suggested that changes in the brain were the most important reason for the decreased requirement of sevoflurane in cirrhotic patients and that BIS values may be a useful quantitative index for recognizing patients with liver dysfunction who may need reduced amount of anesthetic.

Changes of plasma compositions in patients with liver dysfunction can be complex. Here we observed changes in liver enzymes, albumin, bilirubin and coagulation and found a significant increase in ALT and AST in Groups A and B patients, which indicates hepatocyte damages in these patients. Approximately 3 % of sevoflurane absorbed by the body is biotransformed in the liver, through cytochrome P450_2E1_ [31]. Sevoflurane biotransformation decreases when hepatocytes are damaged because the concentration of cytochrome P450 enzymes is reduced in patients and animals with cirrhosis [32, 33]. ALT was negatively correlated with MAC_electric_, which may suggested that the requirement of sevoflurane is associated with hepatocyte function. However, the correlation coefficient was very minuscule (MAC_electric_ = −0.003 ALT +0.053 BIS) and the negative association between ALT and MAC_electric_ cannot be interpreted as clinically relevant.

We used electrical stimulation to determine the sevoflurane MAC as this method has been used previously in humans [21, 34–37]. It has been shown that electrical stimulation is well correlated with the standard noxious incisional stimulus [38]. The electric stimulation of 80 mA at 50Hzused in this study was a supramaximal stimulus with reliable and predictable motor responses. It is noninvasive, repeatable, and requires smaller sample sizes than the classic method. MAC_electric_ is correlated to and less than that determined by the skin incision method [21, 34, 35]. Our data are in normal patients (1.51 % vs. 1.71 %) are consistent with this conclusion and demonstrate that the intensity of the electric stimulus is less than that of the skin incision.

It is important to recognize that although our primary endpoint result showed a significant effect, the sample size is relatively small. Further clinical trials are required to define the relationship between BIS, the level of liver injury, and the MAC of volatile anesthetics. Investigations concerning the mechanism in animal models are also needed.

## Conclusion

In conclusion, MAC_electric_ of sevoflurane in cirrhotic patients was significantly lower than that of patients with normal liver function. The severity of liver dysfunction basing on Child- Pugh grade had no effect on the MAC_electric_ of sevoflurane in cirrhotic patients. ALT and BIS were the risk factors associated the decrease of MAC_electric_. MAC_electric_ could be used by clinicians to guide the best anesthetic practice in this population and beyond. As the results showed, BIS should be recommended to be a routine in monitoring the depth of anesthesia in the patients with liver dysfunction. Furthermore, the BIS values measured before anesthesia could provide a clue of exiting of MHE, which should be given more attention in the anesthetic practice.

## Abbreviations

ALB: Albumin
ALT: Alanine aminotransferase
APTT: Activated partial thromboplastin time
AST: Aspartame aminotransferase
BIS: Bispectral index
BMI: Body mass index
BUN: Urea nitrogen
DBIL: Direct bilirubin
DST: Digit symbol test
ETCO_2_: End-tidal CO_2_ concentrations
FIB: Fibrinogen
HE: Hepatic encephalopathy
HR: Heart rate
IBIL: Indirect bilirubin
IRB: Institutional Review Board
LDH: Lactate dehydrogenase
MAC: Minimum alveolar concentration
MAC_electric_: MAC that prevents movement in response to an electric stimulation
MAP: Mean arterial pressure
MHE: Minimal hepatic encephalopathy
NCT-A: Number connection test A
PT: Prothrombin time
SCr: Serum creatinine
SpO2: Pulse oximetry
TBIL: Total bilirubin
